# Incorporating stand density effects and regression techniques for stem taper modeling of a *Larix principis-rupprechtii* plantation

**DOI:** 10.3389/fpls.2022.902325

**Published:** 2022-09-30

**Authors:** Anyang Xu, Dongzhi Wang, Qiang Liu, Dongyan Zhang, Zhidong Zhang, Xuanrui Huang

**Affiliations:** ^1^College of Forestry, Hebei Agricultural University, Baoding, China; ^2^College of Economics and Management, Hebei Agricultural University, Baoding, China

**Keywords:** taper equations, density factors, nonlinear mixed effects model, nonlinear quantile regression model, *Larix principis-rupprechtii*, plantation

## Abstract

Stem form is the shape of the trunk, differs among tree species and mainly affected by stand density factor. Accurate taper equations are crucial for estimating the stem diameter, form and tree volume, which is conducive to timber utilization and sustainable forest management and planning. Larch (*Larix principis-rupprechtii* Mayr.) is a valuable afforestation species under large-scale development in North China, but no study on the effect of density on its stem taper has been reported yet. The dataset included 396 analytical trees from 132 standard plots of larch plantation in Saihanba, Hebei Province. Based on 12 different forms of models, we explored the optimal basic equation for plantations and the effects of the stand density, basal area, canopy density and different forms of stand density on the prediction accuracy of the variable-exponent models. The variable-exponent taper equation that includes Sd (stand density) was constructed by using nonlinear regression, a nonlinear mixed effect model and the nonlinear quantile regression method. The results indicate that the Kozak’s 2004 variable-exponent taper equation was the best basic model for describing changes in the stem form of larch plantations, and the density factor in the form of $\sqrt{Sd}$ improved the prediction accuracy of the basic model. Among the three regression methods, the quantile regression method had the highest fitting accuracy, followed by the nonlinear mixed effect model. When the quantile was 0.5, the nonlinear quantile regression model exhibited the best performance which provides a scientific basis for the rational management of larch plantations.

## Introduction

The taper equation is one of the most common indicators used to describe the shape of tree boles (Nunes et al., 2010). With a taper curve equation, it is possible to estimate the diameter at the relative height, which corresponds to slenderness and then obtain a stem volume. A high-precision taper equation is crucial for stem profiles (Lumbres et al., 2017), volume estimates (Nunes et al., 2010), sawlog production (Jiang and Liu, 2011), biomass and carbon storage (Koenker and Bassett, 1978; Özçelik and Crecente-Campo, 2016). The variable-exponent taper equation, one of the most extensively used methods in forest properties modeling and analysis, which describe stem shape with a changing exponent or variable from ground to top to represent theneiloid, paraboloid, conic, and several intermediate forms (Kozak, 1988). Its act by changing the independent variable-exponent in the continuous function, better estimates of the tree stem shape can be obtained, and the method can be applied to tree stems of any shape (Zhang et al., 2021). Many studies have indicated that the variable-exponent taper equation is usually subject to less bias and is more precise in estimating diameters at different heights (Kozak, 1988; Muhairwe, 1999). This equation has been widely used for Chinese fir (*Cunninghamia lanceolata* (Lamb.) Hook.), birch (*Betula platyphylla* Suk.), Korean pine (*Pinus koraiensis* Sieb. et Zucc.) and Japanese larch [*Larix kaempferi* (Lamb.) Carr.; Lee et al., 2017; Tang et al., 2017; Zhang et al., 2021].

Most taper equations are determined based on specific tree species, and the fitting accuracy of the model is also affected by tree species, so the taper of various tree species is very different (Muhairwe, 1994). Stem forms varies for different trees due to factors such as stand density (Sharma and Zhang, 2004; Jiang and Liu, 2011; Duan et al., 2016; Sharma, 2020), stand age (Gomat et al., 2011), and crown variables (Valenti and Cao, 1986; Leites and Robinson, 2004; Liu et al., 2020; Liang et al., 2022). However, the influence of stand age and canopy structure variables on the prediction precision of the taper equation in the study of the response of the stem form to various factors is unknown. Most studies (Duan et al., 2016; Sharma, 2020) have considered that the prevailing stand density factor determines the characteristic taper formation among tree species, therefore, it is necessary to add density factor to the taper equation to improve the accuracy of the model. In addition, compared with other stand factors, the density factor is easier to obtain and costs less investigation cost. Jiang and Liu (2011) reported that the stand density indirectly affects the stem form by affecting the crown structure, and a suitable stand density has a good potential for enhancing timber production and wood quality. Currently, stand density indicators include tree per hectare (TPH), basal area (BA), and canopy density (Cd). Additionally, trees per hectare and basal area are the most widely indicators for the stem form (Sharma and Zhang, 2004; Sharma, 2020). For example, Sharma and Zhang (2004) used different forms of plant density (TPH) indicators to describe the stem form of Canadian short-leaved pine (*Pinus banksiana* Lamb.), black spruce [*Picea mariana* (Mill.) B.S.P.] and fir [*Abies balsamea* (L.) (Mill.)] and reported that$\;\sqrt{\mathrm{BA}/\mathrm{TPH}}$ could improve the fit statistics and predictive accuracy for all species. Sharma and Parton (2009) developed a taper equation for jack pine (*Pinus banksiana* Lamb.) and black spruce plantations growing at varying densities, proved BA is the most significant in describing taper. Therefore, including stand density information in modeling tree tapers makes sense.

The variable-exponent taper equation is the main equation used to describe the stem form. Over time, several regression techniques have been reported and evaluated in terms of their accuracy and precision. In the process of constructing the taper equation, nonlinear regression (Lee et al., 2017; Tang et al., 2017), the nonlinear mixed effect model method (Fonweban et al., 2012; Arias-Rodil et al., 2015), and nonlinear quantile regression (Bohora and Cao, 2014; Özçelik et al., 2018; Liu et al., 2020) have been widely used to estimate the parameters of the taper equation of different tree species. However, nonlinear regression requires the assumption of independence and the equal distribution of errors, with a zero mean and constant variance (Demaerschalk, 1973; Sabatia and Burkhart, 2015), but this assumption is violated by temporal correlation and spatial heterogeneity as a result of multiple observations of each tree. A nonlinear mixed effect model can effectively account for this problem. The mixed effect model is a statistical method developed in the late 1900s. The random effect represents the hierarchical structure of the data set and the independent variables can be predicted at different levels. Nonlinear mixed effects modeling approach can improve the goodness-of-fit statistics compared with nonlinear regression (Bronisz and Zasada, 2019; Bouriaud et al., 2019; Amna et al., 2021), it is widely used in forest growth and harvest models such as canopy width model (Yang and Huang, 2017) and single tree growth model (Zhang et al., 2014; Duan et al., 2018; Simone et al., 2020). Compared to traditional regression, nonlinear quantile regression tends to be more efficient and accurate, especially in evaluating values other than the conditional average (Smale et al., 2014). Moreover, this type of model can be used to obtain the regression of the conditional mean and the regression results for arbitrary quantile points, thus providing a wide range of applications, including self-thinning boundary lines (Zhang et al., 2021), diameter growth models (Bohora and Cao, 2014) and tree height diameter models (Rust, 2014).

In different stand types, the density factor, taper equation form and regression techniques play key roles in determining the change in the stem form. However, this method has not been studied independently for a high-precision taper equation based on appropriate density indicators and regression techniques. Larch forests, one of the main plantation types in northeast China, which are an important timber forest species above the middle and high mountains in northern China. It occupies the widest distribution range and the largest stand volume among the main forest ecological tree species in the Yanshan Mountains. The Saihanba Forest Farm in Hebei Province is known for its abundant forest resources. The results of the national continuous forest inventory showed that the forest coverage rate is over 80.0%. It supplies 137 million cubic meters of purified water and 55,000 tons of oxygen to Beijing and Tianjin every year, which is an important ecological barrier to protect Beijing and Tianjin. Larch forests provide essential economic and ecological benefits related to timber production, windbreaks, sand fixation, and carbon storage on the Saihanba Forest Farm. The objectives of this study were (1) to fit the taper equation to provide accurate estimates of the diameter at any height by using 396 analytical data points of larch trees from the Saihanba Forest Farm, (2) to evaluate the taper equations based on different stand density factors to obtain the optimal density factors that affect the taper equation, and (3) to compare the predictive abilities of the nonlinear mixed effect model, nonlinear regression and nonlinear quantile regression to obtain the most flexible and widely applicable regression technique.

## Materials and methods

### Study area

The study area encompassed the Saihanba Forest Farm (41°22′–42°58′ N, 116°53′–118°31′ W) in Hebei Province. The research site is in the warm temperate continental monsoon climate zone. The elevation range of the area is 1,010 ~ 1,940 m above sea level, and the terrain is higher in the north than in the south. The landform is rich and mainly includes plateaus and mountains. The annual average temperature is −1.2°C, and the mean annual temperature ranges from −43.3°C to 33.4°C. The annual precipitation is 452.2 mm, and the annual evaporation is 1,388 mm. The typical soils of the area are aeolian sandy soil, meadow soil, brown soil and gray forest soil. The total operating area is 94,000 hectares, including 73,333 hectares of forest land, of which 57,333 hectares of artificial forests and 16,000 hectares of natural forests; the forest coverage rate is 80%, the total forest volume is 5.025 million m^3^, and the average annual growth rate is 9.7%. The main types of vegetation are grassland, meadow, coniferous and broad-leaved mixed forest, broad-leaved forest, and shrub forest, with a forest coverage rate of 75.5%. The main trees are *Larix principis-rupprechtii* Mayr., *Picea asperata* Mast., and *Betula platyphylla* Suk., and the main shrubs are *Rhododendron micranthum* Turcz., *Syringa oblata* Lindl. var. alba Rehder., and *Sambucus Williamsii* Hance. The main herbaceous plants include *Galium verum* L. and *Menyanthes trifoliate* L.

### Data description

**Figure 1:**
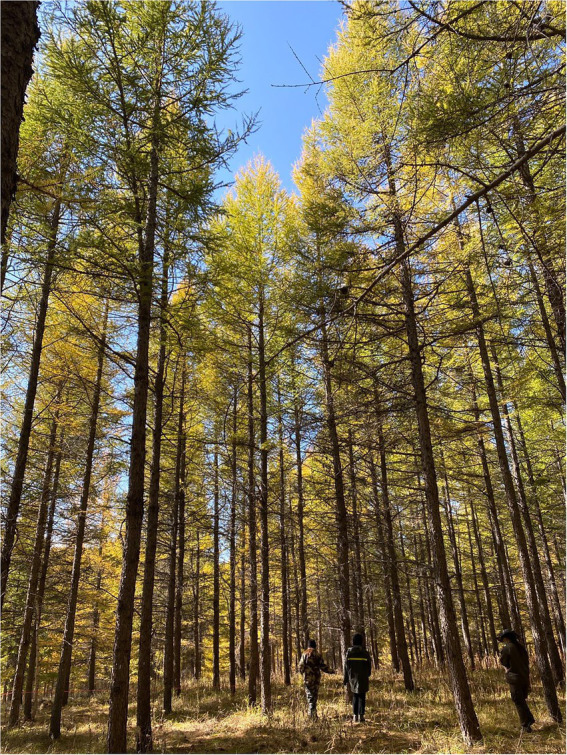
*Larix principis-rupprechtii* Mayr. plantation in Saihanba Hebei Province.

**Figure 2:**
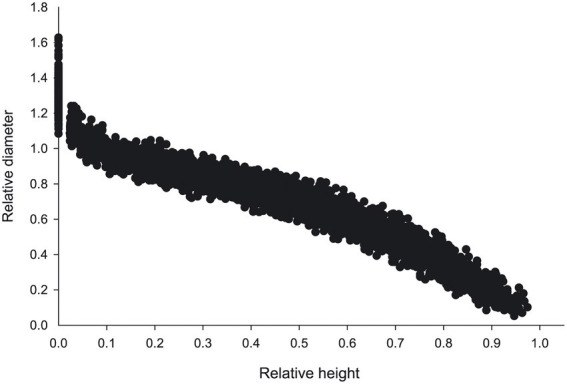
Relative height plotted against the relative diameter of the tree height of *Larix principis-rupprechtii* Mayr. Relative height, ratio of trunk height above ground to total tree height; relative diameter, ratio of diameter to DBH at the height of trunk above the ground.

From 2018 to 2020, 132 sample plots (30 m^2^ × 30 m^2^) in the larch plantation of the Saihanba Forest Farm, Hebei Province (Figure 1). were established. The tree factors (diameter at breast height, tree height, crown width, height under branches), stand factors (age, density, canopy density, basal area) and site factors (altitude, slope, slope position, aspect) of the standard plots were measured. Based on the survey data, three average trees were selected for destructive sampling from each standard plot. The total height H was measured from the stump to the tree tip. The diameters were measured at 5%, 10%, 15%, 20%, 30%, 40%, 50%, 60%, 70%, 80%, and 90% of the total height. From all measured trees, 25% of data were randomly selected as a validation data set, while the rest were used for model fitting. Descriptive statistics of the tree height and diameter at breast height (DBH; Table 1) and the trend of the relative height of the stem form with a relative diameter (Figure 2) are shown.

**Table 1:** Descriptive statistics for fitting and validation data sets of *Larix principis-rupprechtii* Mayr. in study area.

Statistics	Fitting data (*n* = 298)						Validation data (*n* = 98)					
	DBH (cm)	H (m)	Age (years)	Sd (tree·hm^−2^)	Cd	BA (m^2^/hm)	DBH (cm)	H (m)	Age (years)	Sd (tree·hm^−2^)	Cd	BA (m^2^/hm)
Minimum	15.1	10.5	18.0	90.0	0.2	12.8	15.1	10.4	23.0	225.0	0.3	10.6
Maximum	28.8	22.6	59.0	2580.0	0.9	67.7	28.9	22.8	53.0	1845.0	0.9	46.7
Mean.	21.0	17.2	35.9	787.2	0.7	25.3	21.0	17.1	36.7	725.9	0.7	23.4
Std.	3.4	2.5	5.9	396.4	13.6	9.2	3.4	2.6	5.7	368.9	12.6	8.4
CV.	16.3	14.4	16.4	50.4	20.8	36.2	16.3	15.4	15.6	50.8	19.2	35.9

DBH, diameter at breast height; H, total tree height; Age, stand age; Sd, stand density; Cd, canopy density; BA, basal area; hm, hectare; std, standard deviation; CV, coefficient of variation, *n* is the number of trees.

### Basic model

In recent years, researchers have established different forms of taper equations to describe the stem form of different tree species. The taper equation belongs to the empirical equation and the theoretical growth equation, which is a typical nonlinear regression model, and estimation of parameters by nonlinear least square method. The widely used taper equation is generally arranged into three groups: the simple taper equation, the segmented taper function and the variable-exponent taper function (Shahzad et al., 2020). Among them, the simple taper equation has a simple form and estimates the parameters in a straightforward manner, but it has the disadvantage that model fit has large deviation from the data at the bottom of the tree (Kozak et al., 1969; Newberry and Burkhart, 1986; Sharma and Oderwald, 2001). The segmented taper equation states that several polynomials representing different parts of the tree stem are connected through an inflection point, and the tree stem is assumed to have several different geometries from bottom to top. However, the shortcomings are that the formula is too complex, and the parameter estimation method does not converge (Max and Burkhart, 1976; Fang et al., 2000; Brooks et al., 2008). The variable-exponent taper equation has the advantages of a simple structure and the easy convergence of parameter estimation method (Perez et al., 1990; Newnham, 1992; Bi, 2000), and researchers have demonstrated its better fit and suitability for studying the stem form (Özçelik and Crecente-Campo, 2016; Tang et al., 2017). Therefore, this study based on the 12 most widely used forms of variable-exponent taper equations to describe the stem form of larch plantations (Table 2).

**Table 2:** The 12 stem taper equations selected as candidate models.

Origin model form		Parameters	Variables
Kozak (1988)	$d=b_{1}\cdot D^{b_{2}}\cdot b_{3}{}^{D}\cdot {\left[\frac{1-\sqrt{T}}{1-\sqrt{p}}\right]}^{\left(b_{4}T^{2}\cdot {\left(\frac{1.3}{H}\right)}^{4}+b_{5}\ln\left(T+0.001\right)+b_{6}\sqrt{T}+b_{7}e^{T}+b_{8}\cdot \frac{D}{H}\right)}$	b_1_,b_2_,b_3_,b_4_,b_5_,b_6_,b_7_,b_8_,P	D,H,T
Muhairwe et al. (1994)	$d=b_{1}\cdot D^{b_{2}}\cdot b_{3}{}^{D}\cdot {\left[\frac{1-\sqrt{T}}{1-\sqrt{0.01}}\right]}^{\left(b_{4}+b_{5}T^{\frac{1}{4}}+b_{6}T^{\frac{1}{3}}+b_{7}T^{\frac{1}{2}}+b_{8}\mathrm{arc}\;sin(1-T^{\frac{1}{2}}\right)+b_{9}\left[\frac{1}{\frac{D}{H}+T}\right]+b_{10}H}$	b_1_,b_2_,b_3_,b_4_,b_5_,b_6_,b_7_,b_8_,b_9_,b_10_	D,H,T
Muhairwe (1999)	$d=b_{1}\cdot D^{b^{2}}\cdot b_{3}{}^{D}\cdot {\left(1-\sqrt{T}\right)}^{\left(b_{4}T^{2}+\left(\frac{b_{5}}{T}\right)+b_{6}\cdot D+b_{7}\cdot H+b_{8}\cdot \left(D/H\right)\right)}$	b_1_,b_2_,b_3_,b_4_,b_5_,b_6_,b_7_,b_8_	D,H,T
Muhairwe (1999)	$d=b_{1}\cdot D^{b^{2}}\cdot b_{3}{}^{D}\cdot {\left(1-\sqrt{T}\right)}^{\left(b_{3}\cdot T+b_{4}\cdot T^{2}+\frac{b_{5}}{T}+b_{6}\cdot T^{3}+b_{7}\cdot D+b_{8}\cdot \left(D/H\right)\right)}$	b_1_,b_2_,b_3_,b_4_,b_5_,b_6_,b_7_,b_8_	D,H,T
Bi (2000)	$\begin{array}{l} d=D\cdot [\mathrm{logsin}\left(\frac{\pi }{2}\cdot T\right)\\ /\mathrm{logsin}{\left(\frac{\pi }{2}\cdot \frac{1.3}{H}\right]}^{\left[\begin{array}{l} b_{1}+b_{2}\cdot \sin\left(\left(\frac{\pi }{2}\right)\cdot T\right)+b_{3}\cos\left(\left(\frac{3\pi }{2}\right)\cdot T\right)+b_{4}\cdot \frac{\sin\left(\left(\frac{\pi }{2}\right)\cdot T\right)}{T}\\ +b_{5}\cdot D+b_{6}\cdot T\cdot \sqrt{D}+b_{7}\cdot T\cdot \sqrt{H} \end{array}\right]} \end{array}$	b_1_,b_2_,b_3_,b_4_,b_5_,b_6_,b_7_	D,H,T
Kozak (2004)	$d=b_{1}\cdot D^{b_{2}}{\left[\frac{1-T^{1/4}}{1-0.01^{1/4}}\right]}^{\left(b_{3}+b_{4}\left(\frac{1}{e^{\frac{D}{H}}}\right)+b_{5}D\left[\frac{1-T^{\frac{1}{4}}}{1-0.01^{\frac{1}{4}}}\right]+b_{6}{\left[\frac{1-T^{\frac{1}{4}}}{1-0.01^{\frac{1}{4}}}\right]}^{\frac{D}{H}}\right)}$	b_1_,b_2_,b_3_,b_4_,b_5_,b_6_	D,H,T
Kozak (2004)	$\begin{array}{l} d=b_{1}\cdot D^{b_{2}}\cdot H^{b_{3}}\cdot \\ {\left[\frac{1-T^{1/3}}{1-{\left(\frac{1.3}{H}\right)}^{1/3}}\right]}^{b_{4}T^{4}+b_{5}\left(\frac{1}{e^{\frac{D}{H}}}\right)+b_{6}{\left[\frac{1-T^{\frac{1}{3}}}{1-{\left(\frac{1.3}{H}\right)}^{\frac{1}{3}}}\right]}^{0.1}+b_{7}\left(\frac{1}{D}\right)+b_{8}H^{1-T^{1/3}}+b_{9}\left[\frac{1-T^{\frac{1}{3}}}{1-{\left(\frac{1.3}{H}\right)}^{\frac{1}{3}}}\right]} \end{array}$	b_1_,b_2_,b_3_,b_4_,b_5_,b_6_,b_7_,b_8_,b_9_	D,H,T
Lee et al. (2003)	$d=b_{1}\cdot D^{b_{2}}\cdot {\left(1-T\right)}^{b_{3}\cdot T^{2}+b_{4}\cdot T+b_{5}}$	b_1_,b_2_,b_3_,b_4_,b_5_	D.T
Kozak (2004)	$d=b_{1}\cdot D^{b_{2}}\cdot H^{b_{3}}\cdot {\left[\frac{1-T^{1/3}}{1-P^{1/3}}\right]}^{b_{4}\cdot T^{4}+\frac{b_{5}}{e^{D/H}}+b_{6}\cdot {\left[\frac{1-T^{1/3}}{1-P^{1/3}}\right]}^{0.1}+\frac{b_{7}}{D}+b_{8}\cdot H^{1-{\left(\frac{H}{D}\right)}^{1/3}}+b_{9}\cdot \left[\frac{1-T^{1/3}}{1-p^{1/3}}\right]}$	b_1_,b_2_,b_3_,b_4_,b_5_,b_6_,b_7_,b_8_,b_9_,p	D,H,T
Sharma and Zhang (2004)	$d=D\cdot \sqrt{b_{1}\cdot \left(\frac{Z}{H-1.3}\right)\cdot {\left(\frac{h}{1.3}\right)}^{2-\left(b_{2}+b_{3}\cdot T+b_{4}\cdot T^{2}\right)}}$	b_1_,b_2_,b_3_,b_4_	D,H,h,Z,T
Berhe and Arnoldsson (2008)	$d=b_{1}\cdot D^{b_{2}}\cdot {\left(1-\sqrt{T}\right)}^{b_{3}T^{2}+b_{4}\cdot T+b_{5}}\cdot$	b_1_,b_2_,b_3_,b_4_,b_5_	D,T
Sharma and Parton (2009)	$d=D\cdot b_{1}\cdot \left(\frac{Z}{H-1.3}\right)\cdot {\left(\frac{h}{1.3}\right)}^{\left(b_{2}+b_{3}\cdot T+b_{4}\cdot T^{2}\right)}$	b_1_,b_2_,b_3_,b_4_	D,H,h,Z,T

D is the DBH; H is the whole tree height; h is the height of the trunk from the ground; d is the diameter at tree height h; b_i_ and p are the parameters to be estimated; T = h/H; Z = H − h.

### Nonlinear mixed effect model

The mixed model consists of fixed parameters and random parameters, and the random parameters are added to the fixed parameters of the nonlinear model, which vary with changes in different blocks. Zhang et al. (2021) established the variable-exponent taper equation for Chinese fir and found that the nonlinear mixed effect model based on the tree-level effect performed the best. Therefore, in this study, a nonlinear mixed effect model was developed according to tree-level effects. The model expressions are as follows:


(1) $$Y_{ijk}=\int \left(\emptyset_{ijk,}t_{ijk}\right)+\varepsilon_{ijk}$$

(2) $$\begin{array}{l} \emptyset_{ijk}=A_{ijk}\beta +B_{ijk}u_{i}\\ +C_{ijk}u_{ij},u_{i1}\sim N\left(0,\emptyset_{1}\right),u_{i2}\sim N\left(0,\emptyset_{2}\right) \end{array}$$

In equations (1–2), $Y_{ik}$ is the *k*_th_ DBH observation value of the *j*_th_ tree in the *i*_th_ standard plot; $\int \left(\cdot \right)$ contains a differentiable function containing parameter vector $\emptyset_{ijk}$ and adjoint vector $t_{ijk}$; and $\varepsilon_{ijk}$ is the error term which is normally distributed. The expected value is assumed to be zero, and the covariance structure $R_{ij}$ is assumed to be positive; additionally, β is a *n* × 1 dimensional parameter vector, and u_i1_ and u_i2_ are random parameter vectors of tree level that obey an independent normal distribution, with an expected value of zero and variance–covariance structures of ∅_1_ and ∅_2_. Finally, *A_ijk_*, *B_ijk_*, and *C_ijk_* are design matrices.

Determining the random and fixed parameters is the key to establishing a mixed effect model. The inclusion of too many random parameters may lead to problems of overparameterization or nonconvergence. Therefore, in this study, different random parameters were combined separately. The optimal combination of fixed and random parameters was determined by comparing the minimum values of the statistics: Akaike’s information criterion (AIC), Bayesian information criterion (BIC) and twice the negative log-likelihood (−2LL; Scolforo et al., 2019; Liu et al., 2020; Zhang et al., 2021), their expressions are provided at later section (equations 12~15).

Variance–covariance structure can reflect the changes between plots and trees. According to the research of Calama and Montero (2005), the variance–covariance structure of two random parameters was set as a positive definite structure matrix, as follows:


(3) $$D=\left[\begin{array}{cc} \sigma_{1}^{2} & \sigma_{12}\\ \sigma_{21} & \sigma_{2}^{2} \end{array}\right]$$

In equation (3), (*i* = 1, 2) is the variance of the random parameters, and $\sigma_{ij}$(*i*, *j* = 1,2, *i*≠j) is the covariance of the random parameters 1 and 2.

The Davidian and Giltinan (2017) method was used to eliminate the intra-group variance–covariance structure in this study. Previous studies have shown that the mixed effect model of the unstructured covariance–variance matrix can explain the autocorrelation between most observations (Yang et al., 2009). The equation is as follows:


(4) $$R_{ij}=\sigma^{2}G_{ij}^{0.5}\Gamma_{ij}G_{ij}^{0.5}$$

In equation (4), $\;\sigma^{2}$ is the residual variance value of the estimation model; $G_{ij}^{0.5}$is a dimensional diagonal matrix used to describe $n_{ij}\times n_{ij}$ variance heterogeneity within groups; and $\Gamma_{ij}$ is an $n_{ij}\times n_{ij}$ dimensional diagonal matrix describing the autocorrelation structure of errors within a group.

### Nonlinear quantile regression model

The quantile regression estimator, which can estimate the complete conditional distribution of dependent variables and evaluate the influence of different quantiles to predict variables (Koenker and Bassett, 1978), is useful when predicting unexpected conditional means (Weiskittel et al., 2011). Compared with the mean regression model of the least-squares procedure, parameters from the quantile regression are obtained by minimizing equation (5):


(5) $$L_{\min}=\underset{y\geq {\hat{y}}_{r}}{\sum }r\left(y-{\hat{y}}_{r}\right)+\underset{y<{\hat{y}}_{r}}{\sum }\left(1-r\right)\left({\hat{y}}_{r}-r\right)$$

where ${\hat{y}}_{r}$ refers to the estimated value at the quantile. The parameters of each taper equation were calculated with quantiles τ= 0.1, 0.2, 0.3, 0.4, 0.5, 0.6, 0.7, 0.8, and 0.9.

Two methods can be used to determine the parameters of quantile regression. In one method, the parameters of each quantile are directly substituted into the test using the same method as that used in nonlinear regression. In the other method, the interpolation method proposed by Cao and Wang (2015) is used to test by combining quantiles. The first method was used to verify the fitting results of quantile regression in this study.

### Model fitting and evaluation

SAS 9.4 was used to estimate the parameters. Specifically, 12 basic models were fitted with the NLIN module, the mixed effect model was fitted with the NLMIXED module, and the nonlinear quantile regression model was fitted with the NLP module. Statistical metrics for evaluating the model included the mean absolute bias (MAB), the root mean square error (RMSE), the mean percentage of bias (MPB), the coefficient of determination (*R*^2^), the adjusted coefficient of determination (*R*_*adj*_^2^), the mean deviation (Bias), Akaike’s information criterion (AIC), and Bayesian information criterion (BIC; Equations 6~15). These expressions are shown as follows:


(6) $$R^{2}=1-\left[\frac{\sum_{i=1}^{n}{\left(y_{i}-\hat{y_{i}}\right)}^{2}}{\sum_{i=1}^{n}{\left(y_{i}-\bar{y_{i}}\right)}^{2}}\right]$$

(7) $$R_{adj}{}^{2}=1-\left(1-R^{2}\right)\left(\frac{n-1}{n-\lambda }\right)$$

(8) $$MAB=\frac{\sum_{i=1}^{n}\left|y_{i}-{\hat{y}}_{i}\right|}{n}\;$$

(9) $$RMSE=\sqrt{\frac{\sum_{i=1}^{n}{\left(y_{i}-\hat{y_{i}}\right)}^{2}}{n-1}}$$

(10) $$MPB=100\times \frac{\sum_{i=1}^{n}\left|y_{i}-{\hat{y}}_{i}\right|}{\sum_{i=1}^{n}y_{i}}$$

(11) $$\;Bias=\frac{\sum_{i=1}^{n}\left(y_{i}-{\hat{y}}_{i}\right)}{n}$$

(12) $$\;AIC=-2\ln\left(L\right)+2\lambda$$

(13) $$BIC=-2\ln\left(L\right)+\lambda In\left(m\right)$$

(14) −2LL=−2In(L)

(15) $$In\left(L\right)=\log\left(\frac{\sum_{i=1}^{i=n}{\left(y_{i}-{\hat{y}}_{i}\right)}^{2}}{n}\right)$$

where *y_i_* is the measured value, $\hat{y_{i}}$ is the predicted value of the model, $\hat{\bar{y}}=\sum \hat{y_{i}}/n$, $S_{\bar{y}}=\sqrt{\frac{\sum {\left(y_{i}-\hat{y_{i}}\right)}^{2}}{n\left(n-p\right)}}$, *n* is the number of samples, L is the maximum likelihood value, λ is the number of parameters.

## Results

### Selection of base taper model

Based on the nonlinear least-squares method, 12 different forms of variable-exponent taper equations were fitted and evaluated (Tables 3, 4). The Kozak (2004) variable-exponent had the best results. The adjusted coefficient of determination (*R*_*adj*_^2^), Akaike’s information criterion (AIC), Bayesian information criterion (BIC), mean absolute bias (MAB), root mean square error (RMSE), and mean percentage of bias (MPB) of the equation were 0.9353, 16,005, 16,141, 0.7779, 1.0561, and 5.0147, respectively. Therefore, the Kozak (2004) model was selected as the optimal basic equation to construct the variable exponential taper equation to accurately predict the change law of the stem form on larch plantations.

**Table 3:** Parameter estimates with approximate standard errors for 12 selected basic models.

Model	b_1_	b_2_	b_3_	b_4_	b_5_	b_6_	b_7_	b_8_	b_9_	b_10_	*p*
Kozak (1988)	1.217 2	0.960 3	1.000 1	−0.001 0	−0.010 4	−1.100 3	0.614 9	0.142 1			0.038 6
	(0.167 9)	(0.063 7)	(0.003 0)	(0.097 3)	(0.011 8)	(0.179 5)	(0.103 4)	(0.009 0)			(0.015 9)
Muhairwe et al. (1994)	1.296 3	0.976 5	0.999 4	34.807 2	−31.812 3	5.274 4	−7.378 1	−21.403 4	−0.314 6	−0.001 2	
	(0.173 5)	(0.064 2)	(0.002 9)	(5.120 6)	(9.451 9)	(8.322 0)	(3.902 1)	(3.218 8)	(0.027 5)	(0.000 7)	
Muhairwe (1999)	0.899 5	1.002 6	1.000 1	0.512 5	0.512 5	0.003 5	−0.002 0	0.092 5			
	(0.122 4)	(0.065 8)	(0.003 1)	(0.010 2)	(0.001 7)	(0.001 0)	(0.000 8)	(0.011 0)			
Muhairwe (1999)	1.260 1	0.833 3	−1.397 8	0.820 6	0.219 7	−0.2733	0.008 2	−0.167 5			
	(0.025 9)	(0.005 4)	(0.131 1)	(0.291 5)	(0.023 9)	(0.173 7)	(0.000 5)	(0.010 8)			
Bi (2000)	0.166 4	0.216 6	−0.113 8	0.060 7	0.002 4	−0.127 3	0.111 3				
	(0.004 4)	(0.025 5)	(0.010 9)	(0.007 1)	(0.000 2)	(0.004 8)	(0.005 2)				
Kozak (2004)	1.400 6	0.930 2	0.426 9	0.040 8	0.007 2	−0.451 7					
	(0.034 4)	(0.008 1)	(0.008 6)	(0.028 2)	(0.000 3)	(0.015 2)					
Kozak (2004)	1.235 0	1.049 5	−0.059 7	0.265 2	−0.156 6	0.325 1	0.273 6	0.093 5	−0.053 6		0.038 6
	(0.151 6)	(0.051 9)	(0.053 3)	(0.038 7)	(0.213 9)	(0.195 7)	(0.285 5)	(0.075 6)	(0.050 9)		(0.015 9)
Lee et al. (2003)	1.564 4	0.915 4	2.850 8	−3.737 6	1.941 1						
	(0.031 4)	(0.006 5)	(0.062 0)	(0.078 0)	(0.025 7)						
Kozak (2004)	1.005 1	0.928 1	0.081 4	0.525 3	−0.563 0	0.491 8	1.473 7	0.012 3	−0.108 5		
	(0.025 2)	(0.007 4)	(0.009 6)	(0.010 4)	(0.037 4)	(0.011 1)	(0.262 2)	(0.001 4)	(0.015 5)		
Sharma and Zhang (2004)	0.865 9	1.875 9	0.282 2	0.036 8							
	(0.042 6)	(0.018 9)	(0.013 8)	(0.018 9)							
Berhe and Arnoldsson (2008)	1.664 8	0.915 7	1.189 4	−1.210 3	0.794 1						
	(0.028 7)	(0.005 6)	(0.023 2)	(0.026 7)	(0.008 7)						
Sharma and Parton (2009)	0.060 2	−0.191 0	0.599 0	−1.141 6							
	(0.000 2)	(0.006 2)	(0.028 9)	(0.029 9)							

In parentheses is the standard error; *p* are the parameters to be estimated.

**Table 4:** Fit statistics of the 12 selected basic models.

Model	MAB	RMSE	MPB	AIC	BIC	*R* ^2^	*R*_*adj*_^2^
Kozak (1988)	0.783 2	1.085 3	5.056 3	16,075	16,171	0.930 1	0.930 0
Muhairwe et al. (1994)	0.985 4	1.318 3	6.360 4	16,172	16,245	0.921 7	0.921 6
Muhairwe (1999)	0.741 5	0.861 4	5.004 6	21,214	21,254	0.899 4	0.899 3
Muhairwe (1999)	0.738 3	0.859 6	4.981 5	20,311	20,362	0.899 6	0.899 5
Bi (2000)	0.768 6	0.876 7	5.182 6	23,312	23,577	0.887 6	0.887 5
Kozak (2004)	1.097 1	1.404 5	7.081 4	19,210	19,256	0.916 5	0.916 4
Kozak (2004)	0.935 7	1.266 5	6.034 0	16,114	16,187	0.924 7	0.924 6
Lee et al. (2003)	0.973 8	1.281 0	6.277 6	18,209	18,248	0.923 8	0.923 7
Kozak (2004)	0.777 9	1.056 1	5.014 7	16,005	16,141	0.935 4	0.935 3
Sharma and Zhang (2004)	1.213 6	1.584 9	7.831 3	20,520	20,553	0.904 7	0.904 6
Berhe and Arnoldsson (2008)	0.823 7	1.099 9	5.309 4	16,535	16,575	0.923 3	0.923 2
Sharma and Parton (2009)	1.736 6	1.318 8	11.714 0	28,965	29,016	0.841 6	0.841 5

### Variable-exponent taper equation including the density factor

The Kozak (2004) variable-exponent taper equation can reflect the variation pattern of the stem diameter with the tree height. Therefore, three stand density indexes, the stand density (Sd), the basal area (BA) and the canopy density (Cd), and six different forms of Sd were selected to construct the variable-exponent taper equation of larch plantations in the study. The effects of the three stand density indexes and different forms of Sd on the fitting accuracy of the variable-exponent taper equation of larch plantations were evaluated (Table 5). The result show that $\sqrt{\mathrm{Sd}}$was the best stand density function that explained the variation in taper for this tree species, and the variable-exponent taper equation (equation 16) with the stand density factor was constructed. The adjusted coefficient of determination (*R*_*adj*_^2^), mean absolute bias (MAB), root mean square error (RMSE), and mean percentage of bias (MPB) of the equation were 0.9460, 0.7670, 1.0420, and 4.9520, respectively.


(16) $$\begin{array}{l} d=b_{1}\cdot D^{b_{2}}\cdot H^{b_{3}}\cdot \\ {\left[\frac{1-T^{1/3}}{1-X^{1/3}}\right]}^{b_{4}T^{4}+b_{5}\left(\frac{1}{e^{\frac{D}{H}}}\right)+b_{6}{\left[\frac{1-T^{\frac{1}{3}}}{1-X^{\frac{1}{3}}}\right]}^{0.1}+b_{7}\left(\frac{1}{D}\right)+b_{8}H^{1-T^{1/3}}+b_{9}\left[\frac{1-T^{\frac{1}{3}}}{1-X^{\frac{1}{3}}}\right]+b10/\sqrt{sd}} \end{array}$$

**Table 5:** Parameter estimation and fitting statistics of different density factor taper equations.

Parameters	b_10_*BA	b_10_*Cd	b_10_/Sd	b_10_*Sd	b_10_/Sd^2^	b_10_/$\sqrt{Sd}$	b_10_/log(Sd)	b_10_/$\sqrt[{3}]{Sd}$
b_1_	1.003 4 (0.034 1)	1.000 3 (0.026 9)	1.033 9 (0.026 0)	1.063 2 (0.027 6)	1.036 9 (0.026 5)	0.991 3 (0.025 2)	0.969 4 (0.025 0)	1.063 5 (0.030 3)
b_2_	0.929 3 (0.009 6)	0.930 2 (0.007 3)	0.900 2 (0.007 9)	0.905 6 (0.007 8)	0.908 8 (0.007 9)	0.922 7 (0.007 7)	0.908 0 (0.007 5)	0.916 7 (0.007 8)
b_3_	0.080 7 (0.012 2)	0.077 7 (0.009 9)	0.091 7 (0.009 6)	0.093 0 (0.009 5)	0.087 6 (0.009 6)	0.068 8 (0.009 7)	0.091 8 (0.009 7)	0.086 3 (0.009 5)
b_4_	0.522 5 (0.013 3)	0.549 2 (0.025 9)	0.587 3 (0.013 7)	0.481 8 (0.010 6)	0.544 9 (0.011 2)	0.662 5 (0.019 6)	0.691 3 (0.024 7)	0.451 7 (0.015 7)
b_5_	−0.560 0 (0.046 2)	−0.564 6 (0.042 4)	−0.600 3 (0.039 3)	−0.5548 (0.035 2)	−0.580 5 (0.037 9)	−0.787 4 (0.041 7)	−0.630 2 (0.043 1)	−0.532 8 (0.034 3)
b_6_	0.494 5 (0.014 1)	0.498 1 (0.012 6)	0.522 7 (0.012 1)	0.503 5 (0.010 6)	0.511 3 (0.011 6)	0.500 1 (0.012 8)	0.528 0 (0.043 1)	0.490 5 (0.010 4)
b_7_	1.425 9 (0.333 4)	1.414 0 (0.295 8)	1.210 5 (0.277 5)	1.131 2 (0.247 3)	1.253 0 (0.266 8)	2.460 7 (0.295 6)	1.395 7 (0.305 8)	1.187 0 (0.240 0)
b_8_	0.012 0 (0.0017)	0.012 0 (0.001 5)	0.011 6 (0.001 4)	0.012 1 (0.001 3)	0.011 9 (0.001 4)	0.009 9 (0.001 5)	0.011 0 (0.001 5)	0.012 3 (0.001 3)
b_9_	−0.115 2 (0.0198)	−0.111 7 (0.017 3)	−0.107 4 (0.016 1)	−0.118 6 (0.014 9)	−0.112 4 (0.015 6)	−0.078 5 (0.016 9)	−0.094 5 (0.017 5)	−0.121 8 (0.014 7)
b_10_	0.000 4 (0.0048)	0.018 0 (0.006 9)	0.971 1 (0.109 4)	−0.000 5 (0.000 1)	11.172 0 (1.711 8)	0.347 0 (0.032 7)	0.243 3 (0.025 5)	−0.010 5 (0.002 1)
MAB	0.775 5	0.775 8	0.76 8 0	0.770 0	0.771 0	0.767 0	0.768 0	0.774 0
RMSE	1.050 9	1.050 3	1.044 0	1.045 0	1.047 0	1.042 0	1.043 0	1.050 0
MPB	5.000 3	5.000 2	4.955 0	4.971 0	4.975 0	4.952 0	4.960 0	4.998 0
*R* ^2^	0.875 7	0.865 4	0.906 0	0.905 9	0.895 8	0.946 1	0.916 0	0.925 7
*R*_*adj*_^2^	0.875 6	0.865 3	0.905 9	0.906 0	0.895 6	0.946 0	0.915 9	0.925 7

In parentheses is the standard error; Sd, stand density; Cd, canopy density; BA, basal area.

In equation 16, d is the diameter (cm) at height h of the stem; D is the diameter at breast height (cm); H is the tree height (m); T is the relative tree height, i.e., h/H; X is 1.3/H; Sd is the stand density (trees/hm^2^); and b_1_, b_2_, b_3_, b_4_, b_5_, b_6_, b_7_, b_8_, b_9_, and b_10_ are the parameters to be estimated.

### Nonlinear mixed effect model

Based on the variable-exponent taper equation of larch plantations, including the optimal stand density factor, the variable-exponent taper equation of the nonlinear mixed effect of larch plantations was constructed to solve the problem in which the parameters of the nonlinear mixed effect variable-exponent taper equation struggle to converge due to an excessive number of parameters. In the selected basic model, there are 55 forms of 1–2 parameters in Equation 16 fitted randomly in pairs or separately, and 34 combinations converged (Table 6). When random effect parameters acted on *b*_6_ and *b*_8_, the equation showed the best results. The AIC, BIC and -2LL values were 13,766, 13,818 and 13,738, respectively. The parameter estimates of the nonlinear mixed effect variable-exponent taper equation are shown in Table 7.

**Table 6:** Statistics of the AIC, BIC and −2LL for Kozak (2004) variable-exponent taper models with the density effect.

Mixed parameters	AIC	BIC	−2LL	Mixed parameters	AIC	BIC	−2LL
b_1_	15,137	15,188	15,109	b_2_,b_5_	15,143	15,199	15,113
b_2_	15,137	15,182	15,113	b_2_,b_7_	15,143	15,199	15,113
b_3_	15,140	15,140	15,116	b_3_,b_5_	14,112	14,164	14,084
b_4_	15,137	15,181	15,113	b_3_,b_10_	14,199	14,251	14,171
b_5_	14,817	14,862	14,793	b_4_,b_6_	14,424	14,475	14,396
b_6_	14,843	14,887	14,819	b_4_,b_9_	14,637	14,689	14,609
b_7_	14,778	14,823	14,754	b_4_,b_10_	14,272	14,323	14,244
b_8_	15,640	15,685	15,616	b_5_,b_6_	14,409	14,461	14,381
b_9_	15,554	15,598	15,530	b_5_,b_8_	13,834	13,886	13,806
b_10_	14,670	14,714	14,646	b_5_,b_9_	13,896	13,948	13,868
b_1_,b_2_	63,145	63,196	63,117	b_6_,b_8_	13,766	13,818	13,738
b_1_,b_4_	14,285	14,337	14,257	b_6_,b_9_	13,801	13,852	13,773
b_1_,b_5_	14,103	14,155	14,075	b_6_,b_10_	13,896	13,948	13,868
b_1_,b_6_	14,090	14,141	14,062	b_7_,b_9_	13,831	13,883	13,803
b_1_,b_7_	14,065	14,117	14,037	b_8_,b_9_	14,746	14,797	14,718
b_1_,b_9_	45,779	45,831	45,751	b_8_,b_10_	14,032	14,084	14,004
b_1_,b_10_	14,194	14,246	14,166	b_9_,b_10_	14,008	14,060	13,980

AIC, Akaike information criterion; BIC, Bayesian information criterion; LL, Log likelihood.

**Table 7:** Parameter estimates and variance components for the best combinations b_6_ and b_8_ of the nonlinear mixed effect model.

Parameters	Estimate	standard error	95% confidence limits		Value of *p*
b_1_	0.936 2	0.017 9	0.900 8	0.971 2	<0.0001
b_2_	0.939 3	0.006 4	0.926 8	0.951 8	<0.0001
b_3_	0.078 5	0.007 3	0.064 1	0.092 9	<0.0001
b_4_	0.624 1	0.017 6	0.589 7	0.658 9	<0.0001
b_5_	−0.653 4	0.079 0	−0.806 7	−0.495 9	<0.0001
b_6_	0.524 7	0.025 8	0.474 6	0.576 2	<0.0001
b_7_	1.630 2	0.535 6	0.550 5	2.658 7	0.003
b_8_	0.014 0	0.001 4	0.011 3	0.016 6	<0.0001
b_9_	−0.124 7	0.016 4	−0.156 9	−0.092 2	<0.0001
b_10_	0.257 8	0.030 5	0.197 7	0.317 9	<0.0001
Var(*u*_1_)	2.607 1	0.246 5	2.119 6	3.089 9	<0.0001
Var(*u*_2_)	−0.106 5	0.011 9	−0.129 9	−0.083 2	<0.0001
Cov(*u*_1_,*u*_2_)	0.007 7	0.000 8	0.006 2	0.009 2	<0.0001
$\sigma^{2}$	0.579 1	0.011 8	0.555 9	0.602 2	<0.0001

*u*_1_ and *u*_2_ are random parameter vectors; Var(*u*_1_) and Var(*u*_2_) are variances for the random effects u_1_ and *u*_2,_ respectively; Cov(*u*_1_,*u*_2_) is covariance between random effects. $\sigma^{2}$ is residual variance.

### Nonlinear quantile regression model

**Figure 3:**
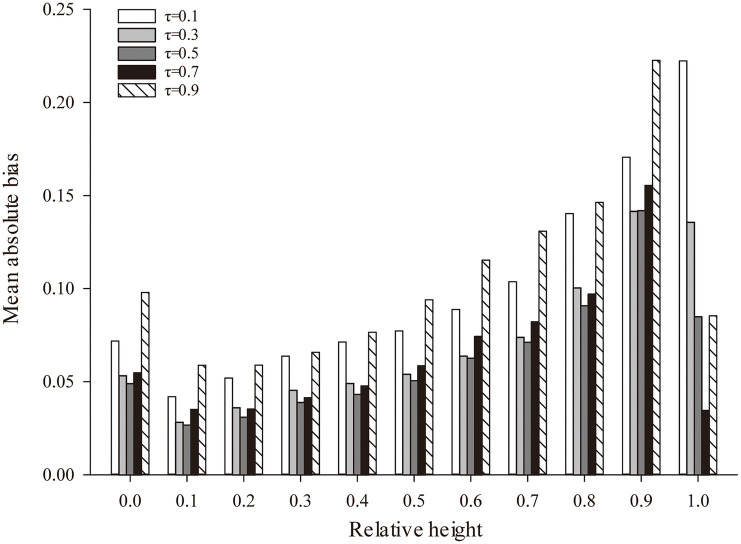
The fitting results of different positions of the stem based on the different quantiles. τ, nonlinear quantile regression (NQR) across nine different quantiles.

Using nonlinear quantile regression technology, the variable-exponent taper equation of larch plantations, including the optimal stand density factor, was constructed. Table 8 presents the estimated parameters of the variable-exponent taper equation of larch plantations at different quantiles (τ = 0.1, 0.2, 0.3, 0.4, 0.5, 0.6, 0.7, 0.8, 0.9). With changes in the stem form, different quantile points had different prediction accuracies for the diameter at different trunk positions (Figure 3). When quantile τ= 0.5, the quantile regression model produced the best MAB value (0.7636). When τ = 0.3, the prediction accuracy was the highest at a relative tree height of 0.9, and the MAB was 0.8470. Therefore, when the quantile τ= 0.5, the variable-exponent taper equation of larch plantations with the optimal stand density factor had the highest fitting accuracy. Among them, the *R*_*adj*_^2^ value (*R*_*adj*_^2^ = 0.9766) was the highest, and the RMSE value (RMSE = 1.0367), MPB value (MPB = 4.9282), and MAB value (MAB = 0.7636) were the lowest.

**Table 8:** Fit parameters and statistics of basic models with density factors at different quantiles.

Parameters	τ= 0.1	τ = 0.2	τ = 0.3	τ = 0.4	τ = 0.5	τ = 0.6	τ = 0.7	τ = 0.8	τ = 0.9
b_1_	0.997 3 (0.021 3)	0.998 5 (0.0353)	0.998 2 (0.021 1)	0.999 7 (0.019 3)	1.000 6 (0.0051)	1.001 4 (0.006 3)	1.002 1 (0.492 1)	1.004 3 (0.100 5)	1.007 2 (0.000 6)
b_2_	0.890 9 (0.017 6)	0.998 5 (0.0092)	0.898 3 (0.007 2)	0.900 8 (0.014 5)	0.900 4 (0.039 2)	0.902 2 (0.025 4)	0.903 0 (0.210 5)	0.906 4 (0.004 2)	0.910 5 (0.002 5)
b_3_	0.092 4 (0.005 3)	0.998 5 (0.0581)	0.090 5 (0.002 1)	0.090 8 (0.001 1)	0.092 6 (0.000 6)	0.093 2 (0.003 2)	0.095 3 (0.007 2)	0.096 5 (0.002 7)	0.096 7 (0.009 2)
b_4_	0.684 5 (0.031 8)	0.998 5 (0.1382)	0.664 6 (0.028 3)	0.660 6 (0.009 5)	0.656 2 (0.011 4)	0.653 6 (0.102 7)	0.649 5 (0.005 7)	0.639 6 (0.005 6)	0.623 8 (0.043 1)
b_5_	−0.607 7 (0.004 0)	0.998 5 (0.0141)	−0.617 7 (0.038 2)	−0.621 2 (0.014 4)	−0.624 8 (0.006 5)	−0.626 5 (0.027 3)	−0.629 2 (0.014 2)	−0.634 5 (0.000 7)	−0.640 8 (0.001 3)
b_6_	0.580 6 (0.010 3)	0.998 5 (0.0154)	0.547 3 (0.003 3)	0.537 5 (0.021 4)	0.530 9 (0.008 4)	0.520 3 (0.023 9)	0.514 5 (0.000 5)	0.500 2 (0.237 2)	0.483 7 (0.006 9)
b_7_	1.280 4 (0.009 2)	1.279 8 (0.0337)	1.278 6 (0.130 3)	1.278 0 (0.063 8)	1.277 0 (0.364 1)	1.277 6 (0.683 5)	1.277 4 (0.050 3)	1.276 2 (0.758 1)	1.274 9 (0.045 1)
b_8_	0.005 6 (0.000 2)	0.007 5 (0.000 6)	0.009 4 (0.000 3)	0.010 2 (0.001 3)	0.011 1 (0.000 1)	0.012 1 (0.004 8)	0.013 8 (0.000 9)	0.015 5 (0.001 1)	0.018 3 (0.006 2)
b_9_	−0.101 3 (0.077 1)	−0.099 2 (0.006 3)	−0.099 2 (0.002 6)	−0.100 6 (0.031 8)	−0.100 4 (0.093 1)	−0.101 4 (0.008 5)	−0.100 7 (0.063 4)	−0.097 4 (0.007 2)	−0.092 4 (0.000 8)
b_10_	0.309 6 (0.017 1)	0.317 4 (0.023 3)	0.321 2 (0.004 6)	0.324 2 (0.010 1)	0.326 5 (0.006 3)	0.328 5 (0.045 7)	0.329 6 (0.008 9)	0.333 5 (0.003 5)	0.339 5 (0.010 7)
MAB	1.191 4	0.968 1	0.847 0	0.782 4	0.763 6	0.785 6	0.849 5	1.015 6	1.334 2
RMSE	1.528 0	1.299 4	1.163 5	1.079 6	1.036 7	1.053 7	1.110 4	1.281 7	1.615 7
MPB	7.686 2	6.251 1	5.469 3	5.046 2	4.928 2	5.066 7	5.478 4	6.554 8	8.610 8
*R* ^2^	0.948 3	0.962 7	0.970 1	0.974 3	0.976 6	0.975 5	0.972 8	0.963 7	0.942 3
*R*_*adj*_^2^	0.948 4	0.962 8	0.970 1	0.974 3	0.976 6	0.975 5	0.972 8	0.963 8	0.942 4

In parentheses is the standard error;τ is different quantiles.

### Model fitting and evaluation

The variable-exponent taper equation proposed by Kozak (2004) was tested and evaluated using 98 data points (Table 9). The variable-exponent taper equation, including the stand density factor, had the highest prediction accuracy. The variable-exponent taper equation of larch plantations, including the stand density factor, was constructed by using the nonlinear least-squares method, the nonlinear mixed effect model and nonlinear quantile regression technology, and nonlinear quantile regression (τ = 0.5) had the highest prediction accuracy.

**Table 9:** Goodness-of-fit statistics of Kozak (2004) for the four different forms using validation data.

Parameters	NR	Contain Sd		
		NR	NLME	NQR(τ = 0.5)
Bias	0.066 4	0.048 5	0.004 1	0.003 2
MAB	0.796 3	0.785 7	0.782 2	0.773 8
MPB	5.145 5	5.077 1	4.758 4	4.497 9
*R* ^2^	0.932 9	0.947 8	0.960 9	0.974 7
*R*_*adj*_^2^	0.932 6	0.947 5	0.960 7	0.974 6

NR, nonlinear regression; NLME, nonlinear mixed effect model; NQR, nonlinear quantile regression;τ is different quantiles.

**Figure 4:**
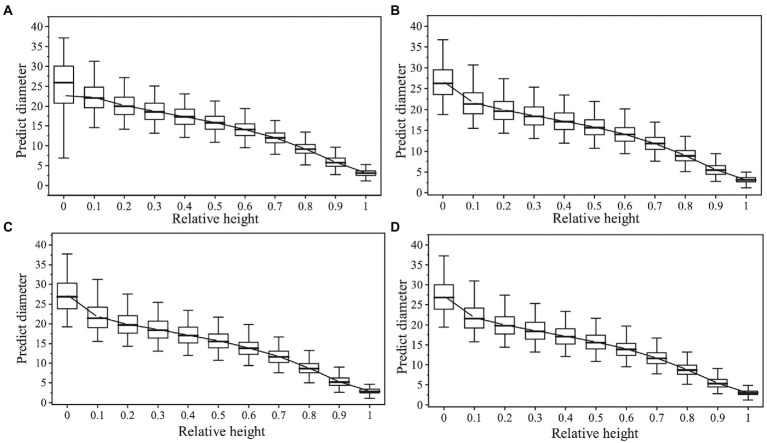
Box plot of the predicted diameter against the relative tree height by using the Kozak (2004) model. **(A)** Nonlinear regression model without density factor; **(B)** nonlinear regression model with density factor; **(C)** nonlinear mixed effect model with density factor; **(D)** quantile regression model with density factor.

Based on the optimal basic variable-exponent taper equation, nonlinear regression, the mixed effect model and nonlinear quantile regression, including the stand density factor, the effects of the density factor and the different parameter estimation methods on the prediction accuracy of the equation were compared (Figure 4). The prediction errors of the Kozak (2004) variable-exponent taper equation with the density factor constructed based on different parameter estimation methods were different. Among them, the variable-exponent taper equation based on nonlinear regression and the nonlinear mixed effect model method had a lower prediction accuracy in the upper part of the trunk (0.7 < h/H ≤ 1). The prediction accuracy for the diameter in the middle and lower parts (0.1 < h/H ≤ 0.6) was higher, but the nonlinear mixed effect model had a higher prediction accuracy. In the nonlinear quantile regression equation, when quantile τ = 0.5, the variable-exponent taper equation, including the stand density factor, could accurately predict the diameter at any relative height of the stem and had the highest prediction accuracy. The smoothness of the fitting curve was closest to the true stem form of the tree.

## Discussion

This study aimed to determine how stand density effects and regression techniques affect the accuracy of taper models. To this end, the optimal basis model were selected from 12 different forms of variable-exponent models and compared the goodness-of-fits of three regression techniques.

### Selection of the optimal basic model

The variable-exponent taper equation is critical for providing a more accurate estimate of the stem form. In this study, 12 different forms of variable-exponent taper equations (Table 2) were fitted to the larch plantation data, and the fitting precision of the Kozak (2004) variable-exponent taper equation was higher than that of the others (Table 4), which is consistent with the conclusions of previous studies (Özçelik and Crecente-Campo, 2016; Liu et al., 2020; Shahzad et al., 2020; He et al., 2021). The Kozak (2004) variable-exponent taper equation well describes the stem form of larch (*Larix gmelinii*), lebanon cedar (*Cedrus libani* A. Rich.) and other tree species and can be used to estimate the volume (Kozak, 2004; Warner et al., 2016). However, other researchers have reached different conclusions. Lumbres et al. (2017) developed a model for Japanese cedar (*Cryptomeria japonica* D.Don) and noted that the Kozak (1988) equation could accurately describe the stem form. Tang et al. (2017) reported that the prediction accuracy of the (Muhairwe, 1999) variable-exponential taper equation was higher than that of the Kozak (1988) equation for *Betula alnoides*. Bi (2000) obtained a fitting accuracy similar to that reported by Kozak (1988) and Bi (2000) at the lower part of the trunk, but the value was significantly higher than that of Kozak (1988) at the top. This phenomenon is related to differences in the tree size. One of the most likely explanations is the ecophysiology of trees, for which the appropriate taper equation for different species varies significantly.

### Effects and selection of stand density variables

The stem form differs among tree species and is influenced by factors such as the site conditions and the stand density (Sharma and Parton, 2009; Jiang and Liu, 2011). In Larson (1963), found that most variations in the stem form are affected by the size of the live crown and the length of the branch-free bole. Burkhart and Walton (1985) first related the crown ratio to parameter estimates in a loblolly pine (*Pinus taeda* L.) taper model and concluded that including the crown ratio as a predictor variable in a taper model was not warranted. Liang et al. (2022) incorporated different crown variables or their combinations into a taper model for a *Cunninghamia lanceolata* forest, and the results confirmed that the accuracy of the taper model incorporating different crown variables was improved. Leites and Robinson (2004) postulated that the operational costs involved in measuring the crown dimensions of standing trees may limit their use. The stand density is a widely used density metric that can be obtained without significant cost. In addition, it is the main factor affecting changes in the stem form (Sharma and Parton, 2009). The results of our study show that the fitting statistics and predictive precision were improved when a stand density factor of b_10_/$\sqrt{\mathrm{Sd}}$ (Table 5) was included in the main model. A similar result was reported by Duan et al. (2016), who incorporated the optimal taper equation of Chinese fir based on the Kozak (2004) model. Sharma and Zhang (2004) introduced a density index $\sqrt{\left(\mathrm{BA}/\mathrm{TPH}\right)}$ into a variable-exponent taper model, which improved the model prediction accuracy when applied to a Korean pine plantation. One possible reason for this inconsistent result is the effect of the tree species and stand type on the stand density (Sharma, 2020). In addition, Sharma and Parton (2009) incorporated BA into a variable-exponent taper model for jack pine and black spruce plantations. Sharma (2020) established the variable-exponent taper equation for Korean pine plantations by combining TPH and BA. In fact, stand density factors inhibited taper increases to a certain extent, affecting the prediction applicability of the taper equation (Jiang and Liu, 2011). However, Duan et al. (2016) noted that this effect mainly affected the stem form below 10% of the tree height, and there was little difference in the prediction of the middle section (Sharma and Zhang, 2004). Nevertheless, density indicators affect individual tree growth and the stem form; therefore, including stand density information in modeling tree tapers makes sense.

### Effects of regression techniques

In most cases, different regression techniques affect the prediction accuracy of the taper model (He et al., 2021). Although nonlinear regression is the most commonly used method, the data used in this method must meet the assumptions of independent error terms (Yang et al., 2009). However, the nonlinear mixed effect model and the quantile regression model can effectively solve these problems. In this study, nonlinear mixed effect variable-exponent taper models were developed, and random effects acting at *b*_6_ and *b*_8_ significantly improved the fit statistics (Tables 6, 7). Similar results have also been presented by Duan et al. (2016), who created a variable-exponent taper equation for Chinese fir. Although the calibrated nonlinear mixed effect model performed better than the nonlinear regression in predicting the stem form, the nonlinear mixed effect method accuracy highly relied on the sampling size and strategy. In previous studies, excessively large or small trees have been confirmed to not be conducive to improving model accuracy (Subedi et al., 2011; Fu et al., 2017; Kb and Lm, 2020). Crecente-Campo et al. (2010) stated that the only method that maintains bias at a low level is random selection. In addition, as the number of measurements included in a subsample increases, the prediction accuracy of the model increases. However, a large sample is often unreasonable because of the increased cost of sampling (Castedo Dorado et al., 2006). Liang et al. (2022) found that a mixed effect model achieved a high accuracy by adding random factors to some parameters, while the requirement of additional measured diameter information for calibration was often not justifiable. These studies suggest that choosing different sample sizes and decreasing the number of samples required for calibration could provide better model fitting results and increase the precision of estimation when applying the mixed effects model. The quantile regression method should be the most flexible; it can not only predict the relationship between the response variable and independent variables in the conditional mean but also quantify the entire conditional distribution of the response variable (Koenker and Bassett, 1978). Our results show that we were able to successfully apply quantile regression to tree taper modeling. When quantile τ = 0.5 (Table 8; Figure 2), the model performed slightly better than it did for the other quantiles. Our results are similar to those of Cao and Wang (2015), who established the taper equation for loblolly pine plantations and recommended the use of five quantile regression methods for prediction purposes. Miao et al. (2021) indicated that when using quantile regression, the locations where the curves crossed one another should be ignored; this recommendation may have been due to the inability of quantile regression to identify the hierarchical structure of the data. Notwithstanding this disadvantage, the quantile regression method was more flexible and the least biased (Table 9) technique for our dataset. Thus, the quantile regression method technique is recommended.

## Conclusion

The results of this study indicate that the Kozak (2004) model was the optimal basic taper model for larch. In addition, the taper model incorporating the stand density index $\left(\sqrt{\mathrm{Sd}}\right)$ gave the most accurate estimation of the diameter. Quantile regression showed the highest accuracy among the several regression techniques. When τ = 0.5, the quantile regression model could accurately describe the stem from the change law of larch. Our study analyzed the impacts of density factors and regression techniques on the accuracy of the taper equation, which provides a new approach for establishing high-precision taper equations.

## Data availability statement

The original contributions presented in the study are included in the article/supplementary material, further inquiries can be directed to the corresponding authors.

## Author contributions

AX, DW, and QL contributed to the study design and performed the formal analysis. AX performed the software analysis and wrote the first draft of the manuscript. DZ contributed data curation. ZZ and XH contributed to the writing, review, and editing. All authors contributed to the article and approved the submitted version.

## Funding

This work was supported by the National Natural Science Foundation of China (32071759), the Asia Pacific Network for Sustainable Forest Management and Rehabilitation (APFNet) project entitled “Spatiotemporal Characteristics of Forest Ecosystems and Its Ecological Restoration in Saihanba National Nature Reserve (2021P2-CHN)” the Natural Science Foundation of Hebei Province project entitled “Study on afforestation under the *Larix principis rupprechtii* plantation in Saihanba based on near-natural management” (C2020204051) the Natural Science Foundation of Hebei Province, China (C2020204026), and the Hebei Province Key R & D Program of China (22326803D).

## Conflict of interest

The authors declare that the research was conducted in the absence of any commercial or financial relationships that could be construed as a potential conflict of interest.

## Publisher’s note

All claims expressed in this article are solely those of the authors and do not necessarily represent those of their affiliated organizations, or those of the publisher, the editors and the reviewers. Any product that may be evaluated in this article, or claim that may be made by its manufacturer, is not guaranteed or endorsed by the publisher.
